# The relationship between ethical leadership, conscientiousness, and moral courage from nurses’ perspective

**DOI:** 10.1186/s12912-022-00941-y

**Published:** 2022-06-24

**Authors:** Samaneh Pakizekho, Maasoumeh Barkhordari-Sharifabad

**Affiliations:** Department of Nursing, School of Medical Sciences, Yazd Branch, Islamic Azad University, Shohadaye Gomnam Blvd., Safaiyeh, Yazd, 8916871967 Iran

**Keywords:** Ethical leadership, Morality, Leadership, Conscientiousness, Moral courage, Nurse

## Abstract

**Background:**

Nurses’ conscientiousness and moral courage are essential to providing high quality care. Leadership is one of the factors that may be very effective in strengthening these characteristics in nurses. Among leadership styles, the ethical leadership has a special value. This study investigated the relationship between ethical leadership of nursing managers, conscientiousness, and moral courage from the nurses’ perspective.

**Methods:**

In this cross-sectional descriptive study, 180 nurses working in hospitals of Yazd, central Iran, were selected through simple random sampling. Three questionnaires: the “Ethical Leadership”, “Conscientiousness”, and “Moral Courage” were used to collect data. Data were analyzed with SPSS20 using descriptive and analytical statistics.

**Results:**

There was a positive and significant relationship between conscientiousness and moral courage with ethical leadership from the nurses’ perspective (*P* < 0.05). The relationship between conscientiousness and moral courage was also significant (*P* < 0.05). The regression analysis showed that ethical leadership can be considered as a predictor of conscientiousness and moral courage.

**Conclusion:**

The relationship between ethical leadership and conscientiousness and moral courage suggests that nursing managers, by adopting such an approach in leadership, can increase conscientiousness and moral courage in nurses.

## Background

Nurses form a key portion of all health care systems [1], and are known as the main guardians of the ethics of caring for a client [2, 3]. Advances in medical technology, scarcity of resources, as well as uncertain and complex clinical problems are imposing stubborn problems on nurses [4]. It can be said that nursing is a morally complex and challenging profession [5–7], and nurses are exposed to moral distress more than other professions [8, 9]. Nurses’ conscientiousness is an important factor in meeting these challenges and is considered as an indicator of professionalism to provide good patient care [10, 11].

Nurses with conscientiousness are conscious and responsive with positive occupational motivation [12]. Conscientiousness is related to goal orientation [13], task orientation, and adaptive capacity [14], that is considered as the strongest predictor of performance [15–17]. Conscientious nurses have more desire to play a role in different situations [12], and show higher role and extra-role performance [18]. They would likely hold strong values and set challenging goals [19]. Conscientiousness is one of the most important codes of nursing ethics in Iran [10, 20]. Acting on duty, supporting the provision of high quality care, and courageous action can be considered as the characteristics of conscientious nurses [3, 6, 21]. Conscientiousness and responsibility of nurses are essential for doing the right thing. On the other hand, insistence on doing the right thing is one of the basic characteristics of moral courage [22].

Moral courage is one of the fundamental values in the nursing profession [23]. Courage-based behavior includes rational reflection, commitment to professional values and principles, and risk-taking [24]. Moral courage helps nurses to act in accordance with what is morally right [25], and in addition to having good human characteristics, to provide acceptable care to the patient, family, and community [26]. Moral courage leads to provision of safe and professional care to clients [27, 28], sensitivity to suffering of others, action to reduce it, and empathy and love [26]. Adequate moral courage enables nurses to challenge unacceptable practices and policies. However, organizations may act defensively in response to the conscientious action of nurses, so that even the most morally courageous nurses may not dare to speak out [29].

Moral courage and conscientiousness are characteristics that are related to individual and organizational factors [10, 30]. Individual factors include socio-demographic factors such as work experience [31, 32], level of education [33, 34], age [33, 35], frequency of challenging situations [31], marital status [32] and so on. As an organizational factor, leadership can be very effective in strengthening the moral courage [36–38], and conscientiousness of employees [39, 40].

Followers’ perspective on leadership is important and evident [41], and affect their performance [42]. According to social learning theory, employees learn by observation of the actions of their leaders. The learning in social learning theory is a cognitive process; that is, learners internalize and make sense of what they observe to reproduce the behavior themselves [43, 44].

Among leadership styles, ethical leadership has a special value and is becoming the most important tool by which leaders can influence people in the organization and their organizational performance [45, 46]. The ethical leadership literature is strongly based on the theory of social learning [47]. Ethical leaders in nursing are individuals who display characteristics such as empathetic interactions, ethical behavior, and exalted manners [48]. Ethical leaders encourage nurses to do the right thing. Also, they have considerable power to create and maintain ethical processes through the ethical climate [49]. Ethical leadership provides the ground for nurses to perform their duties effectively and in a way that satisfies the psychological needs of nurses [50]. Research has shown that ethical leadership can reduce nursing error [51], increase error reporting [51, 52], create organizational commitment [53, 54], improve the health and well-being of employees [55], provide better services [56], and create ethical behavior in employees [56, 57].

Given the issues mentioned above, and considering that conscientious people not only do their task well, but also perform extra-role behaviors such as civic virtue, chivalry, and respect for others [18]; therefore, one of the research hypotheses is that conscientiousness is positively related to nurses’ moral courage. On the other hand, ethical leadership exerts an impact on the behavior and performance of individual employees [47, 52, 58]; besides, followers’ perceptions of their own role and that of others (including the leader) underlie their behaviors and expectations [42]. Therefore, other hypotheses of the research are that ethical leadership has a positive relationship with the conscientiousness and moral courage of nurses from nurses’ perspective.

### Aim

This study examined the relationship between ethical leadership, conscientiousness, and moral courage from nurses’ perspective.

## Methods

### Study design

This descriptive cross-sectional study was conducted in 2020. The study population consisted of all nurses working in hospitals affiliated to Yazd Shahid Sadoughi University of Medical Sciences, Iran. The sample volume was obtained as 178 nurses using the sample volume formula, with a confidence level of less than 5%, test power of 80%, according to the previous similar study [59], and the value of the approximate correlation coefficient of at least r = 0.3. By considering a subject attrition rate of 10%, 200 questionnaires were distributed. Sampling was done by simple random method. Inclusion criteria were having at least a BS degree in nursing, two years of clinical experience, and also willingness to participate in the study. Research tools were distributed in different work shifts and were collected after completion.

### Data collection tools

The required information was collected via four questionnaires: demographic information, ethical leadership in nursing, conscientiousness, and moral courage.

Demographics questionnaire included age, work experience, gender, marital status, and level of education.

The Ethical Nursing Leadership Questionnaire was designed by Barkhordari-Sharifabad et al. in 2017, which evaluates the perception of nurses about the ethical leadership of their managers. This tool measures the level of ethical leadership in nursing managers from nurses’ perspective. This questionnaire entails 49 items and 4 dimensions. The dimensions include ethical-oriented (25 items), pioneering (14 items), power sharing (5 items), and task orientation (5 items). Scoring is based on a five-point Likert scale (very low to very high). Scores range from 49 to 245. A higher score indicates greater ethical leadership. To calculate the weight of each dimension, all scores are converted to a coefficient of 100.

$$\mathrm{Standard}\ \mathrm{score}\ \mathrm{of}\ \mathrm{dimension}\mathrm{s}\ \mathrm{in}\ \mathrm{total}\ \mathrm{score}=\frac{\mathrm{Total}\ \mathrm{score}\ \mathrm{of}\ \mathrm{in}\mathrm{s}\mathrm{trument}\ \mathrm{or}\ \mathrm{each}\ \mathrm{dimension}-\mathit{\min}}{\max -\mathit{\min}}\times 100$$

Therefore, each subscale is rated from 0 to 100, with higher scores indicating higher perceived importance.

This questionnaire was developed using the deductive-inductive approach, and then the face validity, content validity, construct validity, and criterion-referenced validity were assessed [60]. The content validity index (CVI) was obtained as 0.88. An exploratory factor analysis for construct validity showed that this scale consists of four factors [51, 60]. The factor “ethical-oriented” evaluates empathetic interactions, ethical behavior, and exalted manners. As for the “pioneering” factor, the ethical role model and professional insight of the leader are evaluated. The “power sharing” factor includes issues such as empowerment and participation in decision-making. Finally, factor of “task orientation” indicates responsibility, reliability, and accuracy of the leader. Together, these four factors accounted for 72.22% of the total variance of the variables [60]. For criterion-referenced validity, the correlation coefficient between the questionnaire and the ethical leadership questionnaire of Brown et al. [52], as a criterion tool, was examined. This correlation was equal to 0.89, which is significant. The internal consistency reliability and the intra-class correlation coefficient were 0.99 and 0.82, respectively [51, 60].

To measure the rate of conscientiousness, Ardalan and Beheshtirad’s Questionnaire of Conscientiousness (2015) was used [61]. The questionnaire was designed on the basis of Barrick and Mount model (1993) [62] in the form of a 16-item checklist [61]. Eight items (items 1–8) of this questionnaire are related to the dependability subscale and items 9–16 measure the subscale of achievement orientation. The dependability component focuses on being careful, responsible, and maintaining order, while the achievement orientation component helps identify traits related to adopting high standards, striving for excellence, and setting challenging goals [62]. This questionnaire uses a five-point Likert scale (strongly agree to strongly disagree). The minimum possible score will be zero and the maximum 64. A score between zero and 21 indicates a weak conscientiousness, a score between 21 and 32 shows a moderate conscientiousness, and a score above 32 suggests a strong conscientiousness. The results of factor analysis of the questionnaire yielded the two desired dimensions, and its reliability, based on Cronbach’s α coefficient, was 0.84 [61].

The Moral Courage Questionnaire was designed by Sekerka et al. in 2009 [63]. This questionnaire consists of fifteen items and each item is scored with a 7-point Likert scale ranging from “It’s never true” to “It’s always true”. The five dimensions of this questionnaire are moral agency, multiple values, endurance of threat, going beyond compliance, and moral goals. Moral agency is an innate talent, readiness, and desire to show moral behavior and solve moral problems. Multiple values evaluate a combination of personal, professional, and organizational values. Endurance of threat indicates perception and recognition of threat, pressure, and fear by the individual and the ability to withstand and overcome these pressures. Going beyond compliance means that the person is a pioneer in the realization of moral ideals in the organization and tries to promote appropriate moral behaviors in his organization by adopting a preventive approach to immoral actions. Moral goals means setting personal goals based on respect, honesty, and attention to others, preferring the interests of others to oneself, and acting on the basis of moral virtues. Each dimension has three items. The range of scores in each dimension is from 3 to 21 and in total from 15 to 105 [63]. The validity and reliability (Cronbach’s α) of the Questionnaire were 0.81 and 0.85, respectively [64].

### Data analysis

The data were imported into SPSS20 after encryption/coding. A descriptive analysis was performed, with numerical variables described using means, standard deviations, and absolute and relative frequencies. Pearson’s correlation coefficients was used to determine the relationship between ethical leadership, conscientiousness, and moral courage. A multiple regression analysis was conducted to predict factors that could affect the conscientiousness of nurses. Before performing the tests, the normal distribution of the data was checked using Kolmogorov-Smirnov (KS) test (*P* > 0.05).

### Findings

Totally, 180 questionnaires were collected and analyzed. Participants had the mean age and work experience of 33.93 ± 7.61 and 10.21 ± 9.6 years, respectively. The majority of participants held a BS degree (90.6%), were female (58.9%), and were married (65.6%) (Table 1).

**Table 1:** Demographic characteristics of the participants

Variable	Mean ± SD	Frequency (%)
Age	33.93 ± 7.61	
Work Experience	10.21 ± 9.6	
Gender		
Female		106 (58.9)
Male		74 (41.1)
Education level		
B.S.		163 (90.6)
M.Sc.		17 (9.4)
Marital status		
Married		118 (65.6)
Single		62 (34.4)

As it is shown in Table 2, the participants scored moderately on perceptions of their nurse managers’ ethical leadership (180.383 ± 32.278). After weighting the scores according to standard procedures, findings showed that the dimensions of power sharing (68.055 ± 19.168), pioneering (67.539 ± 17.158), task-oriented (67.472 ± 19.975), and ethical-oriented (66.455 ± 16.746) had the highest weight in the ethical leadership score, respectively.

**Table 2:** Descriptive findings of ethical leadership, conscientiousness, and moral courage

Variables	Number of items	Min.-max.	Mean ± SD.	Standard Score (Mean ± SD.) of 100
Ethical-oriented	25	34–125	91.455 ± 16.746	66.455 ± 16.746
Pioneering	14	14–70	51.822 ± 9.608	67.539 ± 17.158
Power sharing	5	5–25	18.611 ± 3.833	68.055 ± 19.168
Task-oriented	5	5–25	18.494 ± 3.995	67.472 ± 19.975
**Ethical leadership**	49	58–245	180.383 ± 32.278	67.032 ± 16.468
Dependability	8	12–30	20.433 ± 3.041	–
Achievement	8	12–30	20.666 ± 3.412	–
**Conscientiousness**	16	25–59	41.100 ± 5.911	–
Moral agency	3	6–21	15.766 ± 3.529	–
Multiple value	3	6–21	15.311 ± 3.379	–
Endurance of threat	3	6–21	15.244 ± 3.461	–
Going beyond compliance	3	6–21	15.566 ± 3.420	–
Moral goals	3	5–21	15.122 ± 3.991	–
**Moral courage**	15	30–105	77.011 ± 15.331	–

The results showed that the majority of nurses (92.2%) were at a strong level in terms of conscientiousness; moreover, the average score of the achievement dimension was slightly higher than dependability. Also, the level of moral courage of nurses was at a high level, wherein the highest average related to the dimension of moral factor and the lowest average related to the dimension of moral goals (Table 2).

The results showed a positive and significant correlation between ethical leadership and the dimensions of task-oriented, power sharing and pioneering with moral courage (*P* < 0.05). Also, Pearson statistical test showed a positive and significant relationship between ethical leadership and all its dimensions except for power sharing with conscientiousness (*P* < 0.05). There was a statistically significant correlation between conscientiousness and moral courage and its dimensions (*P* < 0.05) (Table 3).

**Table 3:** Correlation matrix of ethical leadership, conscientiousness, and moral courage

Variables	1	2	3	4	5	6	7	8	9	10	11	12	13	14
1-Ethical-oriented	1													
2-Pioneering	.894^**^	1												
3-Power-sharing	.761^**^	.848^**^	1											
4-Task-oriented	.780^**^	.813^**^	.778^**^	1										
5-Ethical leadership	.972^**^	.963^**^	.862^**^	.863^**^	1									
6-Moral agency	.176^*^	.230^**^	.176^*^	.181^*^	.203^**^	1								
7-Multiple value	.197^**^	.280^**^	.226^**^	.254^**^	.244^**^	.667^**^	1							
8-Endurance of threat	.121	.185^*^	.136	.139	.151^*^	.751^**^	.735^**^	1						
9-Going beyond compliance	.105	.163^*^	.094	.163^*^	.134	.649^**^	.699^**^	.768^**^	1					
10-Moral goals	.017	.093	.032	.061	.048	.586^**^	.505^**^	.682^**^	.773^**^	1				
11-Moral courage	.139	.217^**^	.150^*^	.181^*^	.177^*^	.844^**^	.828^**^	.910^**^	.901^**^	.833^**^	1			
12-Dependability	.217^**^	.205^**^	.146	.168^*^	.212^**^	.353**	.270^**^	.275^**^	.229^**^	.127	.287^**^	1		
13-Achievement	.213^**^	.162^*^	.068	.111	.180^*^	.390^**^	.287^**^	.312^**^	.283^**^	.184^*^	.334^**^	.677^**^	1	
14-Conscientiousness	.235^**^	.199^**^	.114	.150^*^	.213^**^	.407^**^	.305^**^	.321^**^	.281^**^	.171^*^	.341^**^	.905^**^	.926^**^	1

** Correlation is significant at the 0.01 level (2-tailed)

* Correlation is significant at the 0.05 level (2-tailed)

In the next step, using two separate stepwise regressions analysis, changes in dependent variables (conscientiousness and moral courage) induced by independent variables (ethical leadership and demographic characteristics) were predicted. Ethical leadership was retained as the only predictor that uniquely contributed to conscientiousness and moral courage, and all other variables were excluded. Based on the results presented in Table 4, ethical leadership accounts for 6.5% of the variance in conscientiousness (*R*^*2*^ = .065, *P* = .002) and 4.4% of the variance in moral courage (*R*^*2*^ = .044, *P* = .009).

**Table 4:** Multiple linear regression analysis (stepwise method) for conscientiousness and moral courage

Criterion variable	Model	Predictor	Unstandardized Coefficients		Standardized Coefficients	t	Sig.	95% Confidence Interval for B		R	*R*^*2*^	Adjusted *R*^*2*^
			B	SE	Beta			Lower	Upper			
Conscientiousness	Model 1									.255	.065	.059
		(Constant)	32.386	2.685		12.061	.000	27.080	37.692			
		Ethical leadership	.047	.015	.255	3.229	.002	.018	.076			
Moral courage	Model 1									.211	.044	.038
		(Constant)	58.252	7.090		8.216	.000	44.243	72.261			
		Ethical leadership	.102	.039	.211	2.638	.009	.026	.178			

## Discussion

This study determined the relationship between conscientiousness, and moral courage, and ethical leadership from the perspective of nurses working in the selected hospitals of Yazd, Iran.

There was a statistically significant relationship between moral courage and nurses’ conscientiousness. Studies indicate that conscientiousness increases courage in duty, perseverance, and persistence, and overshadows fear [65, 66]. The results of Abbasi-Asl and Hashemi’s research indicated that conscientiousness, which is one of the dimensions of the five-factor model of personality, is related to moral courage. It is clear that the individual who is responsible and follows the organizational rules and values, has more moral courage, because the basic characteristic of moral courage is to continue to do the right thing [22]. Conscientious nurses feel more responsible for doing good things. They do not hesitate to do anything for the benefit of their clients [67], and act as the patients’ advocates when good care is threatened [68].

The results of this study showed that there was a positive and significant relationship between ethical leadership and nurses’ conscientiousness. Many studies have shown that the ethics-based performance of ethical leaders affects their influence and credibility, and thus enhances the conscientiousness of employees [69]. Also, when the leader is focused on promoting the norms and values of the organization, employees tend to be more involved. This motivates employees to take responsibility for their own duties and increase their engagement in achieving their career goals [70]. Nurses’ conscientiousness guides them in making ethical decisions based on ethical professional codes and guidelines [71, 72]; ethics-based leadership skills play a major role in this regard [47, 70, 73–75].

From the nurses’ point of view participating in the study, the leader’s pioneering and task orientation are related to their conscientiousness. Pioneering means that nursing leaders must not only be moral and conscientious individuals, but they must also go one step further and promote conscientious behaviors through role models [76]. The results of other studies indicate the power of the role model in creating positive consequences for employees and emphasize the importance of studying the role model, in order to better understand the conditions of the impact of ethical leadership on the attitude and behavior of employees [77]. The dimension of task orientation includes such items as the commitment to obeying the rules and regulations and prioritizing the interests of the organization. The task orientation of leaders promotes the willingness of employees to adhere to principles of action and compliance with commitments and policies [78]. Despite the fact that ethical leaders, by sharing power, give employees a sense of competence and merit [79], leading to greater employee responsibility and participation [58], the results of this study showed that there is no relationship between power sharing and conscientiousness. It may be argued that there is no direct relationship between the two and more research is needed to examine the mediating variables between these two factors.

The results also showed that ethical leadership had a positive and significant relationship with moral courage. Because acting morally and courageously can be strenuous, nurses tend to have the support of leaders [25, 68, 80]. Supportive behavior is a characteristic of ethical leadership that helps nurses perform their duties more effectively [48, 51]. Also, the results of a study indicated the effect of moderating role of moral courage in the indirect effect of ethical leadership on the disclosure of suspicious actions (Internal Whistleblowing) [81]. In another study, it was found that ethical leadership and employee loyalty are related to moral courage and that moral courage, as a mediating variable, is relatively involved in the relationship between ethical leadership and employee loyalty [82]. Creating ethical behavior in employees and their ethical guidance is one of the consequences of ethical leadership in nursing [56]. When leaders portray role models of morality and constantly promote and encourage moral values in their followers [53, 83], followers’ motivation is strengthened by moral courage and this increases moral actions.

The results showed that demographic characteristics did not contribute to explaining variance in conscientiousness and moral courage significantly. In line with the results of the present study, the results of Hauhio et al.’s study showed that there was no relationship between age, work experience, and highest degree with moral courage [30]. No significant correlation was found between the level of conscientiousness and job factors in the study by Kwiatosz-Muc et al. [84]. Of course, these findings are inconsistent with some previous research findings [31, 33]. Difference in research units and the cultural and value differences could be the reason for these disparities. Iranian civilization emphasizes the observance of moral behaviors, meritocracy, and so on [57]. In Iranian society, nurses and nursing leaders are mostly Muslim and, regardless of any underlying factors, consider themselves responsible for performing their duties and ethics [10, 48].

### Limitations

Perhaps, the strong point of this research was the use of valid and reliable indigenous tools specific to the nursing profession to measure the level of ethical leadership. However, one of the limitations of this study was the use of self-report tools to collect data, and there was a probability of tiredness and lack of opportunity for completing the questionnaire. As a result, some nurses may not be able to answer the questions correctly. Furthermore, social desirability bias is a problem that should be considered in the use of self-reporting tools. Another limitation was the presence of confounding variables such as the effective factors of nurses’ concentration due to heavy workload, which could not be controlled. In this research, the cross-sectional design has been used. For this reason, it makes causation impossible. Also, because the present study was conducted on selected nurses from one city, the results obtained from this study cannot be generalized to all nurses, thus jeopardizing the external validity of the study.

## Conclusion

The relationship between ethical leadership, conscientiousness, and moral courage suggests that nursing managers, by adopting such an approach in leadership, can increase conscientiousness and moral courage in nurses. Informing nursing managers and providing supportive programs such as periodic counseling can be effective in guiding their performance.

## Data Availability

The datasets generated and analyzed during the current study are not publicly available due to an agreement with the participants on the confidentiality of the data but are available from the corresponding author on reasonable request.

## Abbreviations

MSc: Master of Science
BS: Bachelor of Science
SD: Standard Deviation
ANOVA: Analysis of Variance
KS: Kolmogorov-Smirnov
